# VR Head Tracking as a Useful Tool for the Qualitative Assessment of Cervical Spine Movement

**DOI:** 10.3390/s25072172

**Published:** 2025-03-29

**Authors:** Paweł Sip, Marta Kozłowska, Marcelina Ochowiak, Dariusz Czysz, Przemysław Daroszewski, Przemysław Lisiński

**Affiliations:** 1 Department of Rehabilitation and Physiotherapy, Poznan University of Medical Sciences, 28 Czerwca 1956 Str., No 135/147, 60-545 Poznań, Poland; kozlowskam@ump.edu.pl (M.K.); plisinski@ump.edu.pl (P.L.); 2 The Student Scientific Society, Poznan University of Medical Sciences, Rokietnicka Str., No 5, 60-806 Poznań, Poland; 85370@student.ump.edu.pl; 3 SciTech, Zbąszyńska Str., No 7/7, 60-359 Poznań, Poland; dariusz.czysz@gmail.com; 4 Department of Organization and Management in Healthcare, Poznan University of Medical Sciences, Przybyszewskiego Str., No 39, 60-356 Poznań, Poland; dyrektor@orsk.pl

**Keywords:** cervical spine, Virtual Reality, assessment, movement

## Abstract

Virtual reality (VR), among other technologies, is establishing the direction of development in the rehabilitation field. Diagnostic tools in cervical spine movement assessments have been described in many studies. The aim of this study was to present the potential of using VR for diagnosing movement quality disorders in the cervical spine. Additional objectives of the research include determining whether Virtual Reality conditions are reliable and objective for measuring the range of motion of the cervical spine when using the proprietary VR Head Tracking application, as well as assessing the level of satisfaction and tolerance of this type of diagnostic method among the subjects. We reached the following conclusions: the measurements of the cervical spine range of motion conducted using the proprietary VR Head Tracking application are reliable and objective, the level of satisfaction with the diagnostic method, as well as the tolerance of the VR examination, is **consistently** favorable in adults, the results obtained from the VR application allow for a deeper analysis of the data, beyond just the angles of the range of motion. The use of diagnostic methods in a Virtual Reality environment for assessing the biomechanics of the musculoskeletal system in the cervical spine is valid and can be efficiently and non-invasively carried out.

## 1. Introduction

Technology is quickly permeating medicine [1,2,3]. The further development of research methods in particular clinical disciplines will require the use and integration of advanced technical and informational knowledge in order to create holistic solutions [4,5,6]. This is particularly important in the field of diagnostics and in the treatment of musculoskeletal disorders, as the resulting disabilities determine the quality of life for millions of people. In practical terms, this will lead to increased objectivity in motor system examinations and the repeatability of the obtained examination results while maintaining their high accuracy, which will make the diagnosis more credible in a functional sense. It is also worth noting that the rapid aging of society is leading to a growing number of people with various mobility impairments, which creates a challenge in accessing objective diagnostics and limits physicians’ ability to implement targeted therapy. Implementing network connections and appropriate applications will soon enable automatic, fast, and accurate data gathering, processing, and analysis, as well as diagnostics and the implementation of therapeutic algorithms, which could result in the optimization of working time and allow for a greater focus on treatment. Obviously, it is essential to emphasize the leading role of humans throughout the entire process, despite the emerging concepts surrounding the use of technological solutions such as Virtual Reality (VR) [7,8].

An excellent example of the potential and capabilities of computer science techniques in physiotherapy is dysfunction resulting from various spine pathologies. Range of motion limitations can be both a reason for and a consequence of proximal and distal motor system impairment. The leading symptom concerning the cervical spine is restricted mobility. Precise measurements of mobility are part of the basic functional examination; however, possible compensations in the trunk area can significantly affect the measurement results. Additionally, hypermobility or hypomobility in specific regions of the cervical spine, difficulty in performing movements without undesirable additional components along the axis and plane of motion set by the therapist, and excessive soft tissue tension affecting the spine joints are considered local compensatory mechanisms that significantly influence the accuracy of the measurement [9]. Traditionally, clinical methods such as an inclinometer [10], a goniometer [11], and measuring tape [12,13] are used to measure the movement of the entire spine or its parts. However, these methods have limitations due to the nature of the methodology itself and, as a result, are characterized by significant subjectivity. On the other hand, methods such as three-dimensional measurement systems [14], motion acquisition systems using cameras [15,16], and radiographic imaging for range of motion [15] offer greater measurement accuracy, but they are relatively expensive and require complex measurement procedures. Completely new horizons are opened up by measurement techniques using VR [17], due to their soon-to-be widespread availability, relatively low equipment cost, and, most importantly, the ability to conduct the examination with the maximum elimination of substitution by other body segments during movement. Utilizing VR technology enhances access to physiotherapy while enabling a more effective selection of therapeutic methods. If VR technology becomes more widely accessible, it will create new opportunities for screening and preventive care while enabling a more effective selection of therapeutic methods. The primary goal of increased accessibility is to enhance diagnostics and therapy using interactive tools that allow for faster and more precise analyses due to the repeatability of the given schemes. VR could also be highly beneficial for medical professionals, enabling them to assess specific movements with greater accuracy and data gathering. The immediate availability of results and real-time biofeedback allows for quicker therapeutic decision making. Additionally, VR is well tolerated by patients, making it a comfortable and, most importantly, objective and engaging tool that enhances both the diagnostic and therapeutic experiences. This has the potential not only to improve treatment efficiency but also to ensure more personalized and precisely adjusted therapy methods [18,19,20].

In the context of measuring the range of motion, it is important not only to assess the degrees of mobility or the overall range of motion in a given plane but also to evaluate the movement pattern performed by the subject. The movement pattern, from the starting point to the final position, is defined by the engagement of specific muscles and muscle groups in a predetermined order, utilizing the minimum amount of energy from the body [21]. Evaluating given movement in this way in clinical practice requires the use of electromyographic (EMG) measurements, which assess muscle excitability within specific ranges of motion, indicating physiological motor control [22]. Considering that the basis of movement pattern disorders is typically associated with the nervous system, the accuracy with which movement tasks are executed can therefore also serve as a valuable source of data for analysis in the evaluation process. However, this complex measurement is not widely available, as it requires meeting several conditions and utilizing additional human and time resources [23,24].

The aim of our study is to present the potential application of modern technology implemented in VR for diagnosing movement quality disorders in the cervical spine. Our choice is related to the fact that this part of the musculoskeletal system affects the degree of physical fitness and the basic motor skills of the entire body. Additional objectives of the study include determining whether the conditions of Virtual Reality are reliable and objective for measuring the range of motion of the cervical spine using the proprietary VR Head Tracking application, as well as assessing the level of satisfaction and tolerance of this type of diagnostic method among the subjects.

## 2. Materials and Methods

The study group consisted of students from the Poznan University of Medical Sciences and medical staff from the Wiktor Dega Rehabilitation Hospital in Poznan. A total of 17 women (68%) and 8 men (32%), aged between 22 and 42 years, were examined. The average age was 26 years (min/max 22–42). The following inclusion criteria were defined:

- Age range from 20 to 45 years,
- Absence of current cervical spine pain symptoms,
- No documented medical conditions or injuries in individual medical records that could affect cervical spine functionality, particularly its mobility.

**Figure 1:**
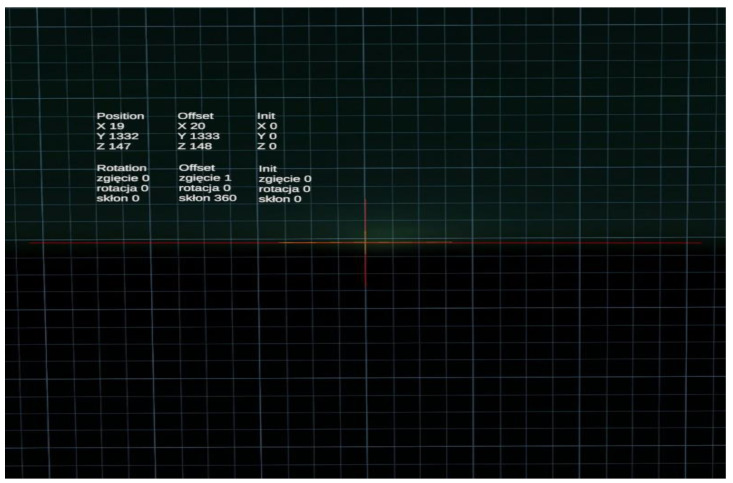
VR Head Tracking application: user’s view.

The research methods included a personal questionnaire and diagnostic custom application VR Head Tracking. The questionnaire consisted of two parts: personal data of the participant, along with a survey assessing the application and the level of satisfaction with the conducted examination. The first part of the questionnaire included five questions regarding demographic data, such as age, gender, and information about the general condition of the musculoskeletal system; this was completed prior to the examination. The second part, completed immediately after the examination, contained 10 questions about the participants’ perception of the VR measurement application and their satisfaction with this type of diagnostic approach. The diagnostic system is built of three parts: a Meta Quest 3 or Meta Quest 2 Pro VR headset, an application installed on the VR headset, a data aggregation system and data exporting program readAwsHead.exe. Meta Quest Virtual Reality headsets are the state-of-the-art stand-alone hardware designed and produced by Meta. They allow a user to immerse themselves in a virtual experience. The headset is mounted on one’s head. Meta Quest offers a pro version of the straps. The comfort relief strap used in the research evenly distributes the weight on the head and relieves pressure on the face, and its corresponding versions positively influence the distribution of the headset’s center of gravity. The 300-degree dial allows for a much better fit adjustment than standard straps. The foam cushion adapts to different head shapes. Two screens built into the headset show the image while the sensors measure the head position and orientation to guarantee the user that the image “moves” with them. The headset includes two hand controllers. However, they are not used in the described application. In our study, the subject does not interact with the environment and there is no need to use the controllers. What the user experiences in the VR Head Tracking application appears as shown in Figure 1.

The head position and orientation can be measured in three ways. External cameras can track the position and rotation of the headset based on recorded images of the environment. Both headsets are equipped with accelerometers that can track the movement of the device. Meta Quest 3 is additionally equipped with a depth sensor that can track the headset’s location in a 3D environment. The application installed on the headset uses the information provided by the Meta SDK API to track and record the position and rotation of one’s head. Detailed information about the programming environment is presented below:
**Unity Version**Unity 2022.3.51f1**Meta SDK Version**Meta XR Core SDK 69.0.1Meta XR Interaction SDK 69.0.2Meta XR Interactions SDK Essentials 69.0.1**Additional Libraries**TextMeshPro 3.0.9AWSSDK.Core.dll 3.7.400.0AWSSDK.TimestreamQuery.dll 3.7.400.0AWSSDK.TimestreamWrite.dll 3.7.400.0

The recorded information is measured 60 times per second. The value of 60 Hz can be read stably in Unity; it is the frequency at which the physics parameters of objects are calculated. In Unity, it is also possible to read at the frame refresh rate, but this value is variable. The data are visualized in front of the user’s eyes and transferred to the server. The application allows the user to set up the initial head position and helps them to conduct straight vertical and horizontal head movements.

All recorded data are also transferred to a remote server where they are aggregated and stored for further processing and analysis. The data are grouped into sessions assigned to a given user and their sequence of moves. The records stored in the database can be used to extract detailed information about the user’s head movement and to visualize it. The data can be also compared between different sessions to track the changes in one’s mobility over time.

**Figure 2:**
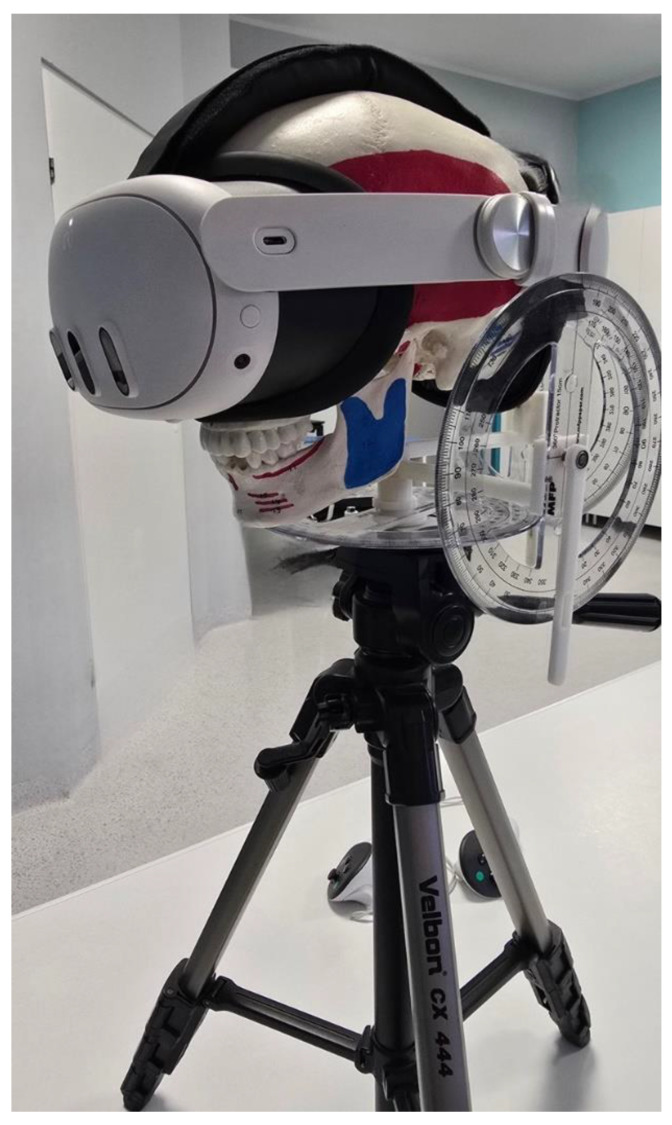
Custom-designed research model.

**Figure 3:**
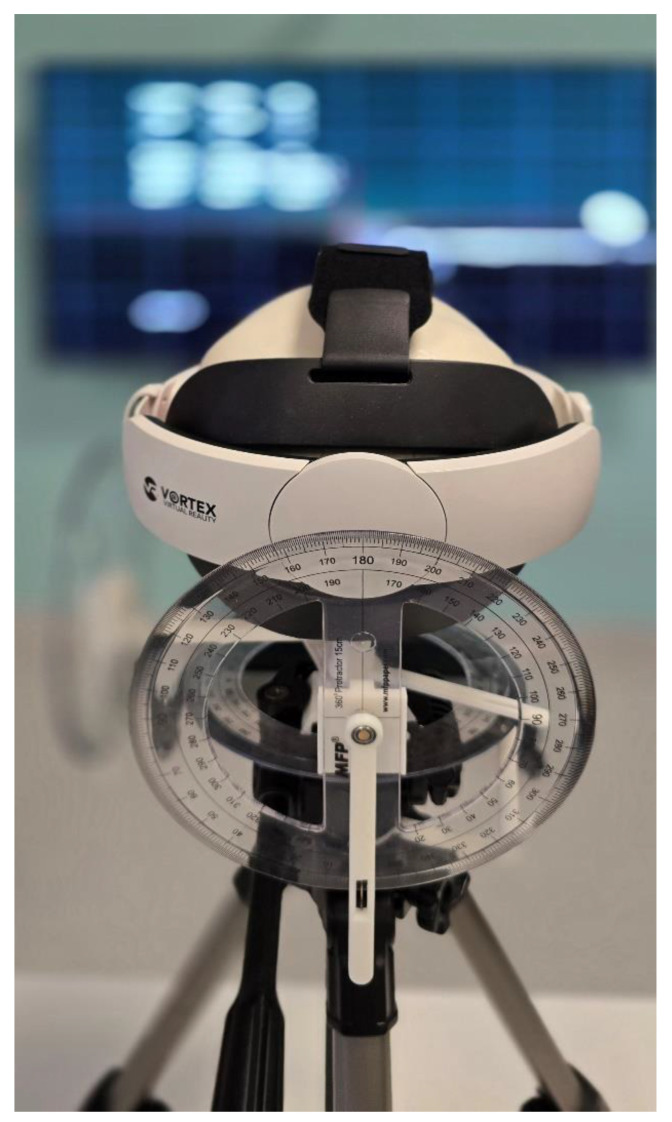
Custom-designed research model.

The research protocol consisted of two parts: the first experiment was conducted without participants and the second involved a user study. To verify the reliability of the measurements conducted by the application, we utilized a custom-designed research model in the first part of the study. This model consisted of a physical skull replica, a stand, and necessary components developed and printed using a 3D printer. The model allowed movement in sagittal, frontal, and transverse planes, mimicking the natural movement of the human head. A VR headset (Meta Quest 3) was mounted on the model. To validate the measurements, the model was equipped with three calibrated goniometers. The analog measurements of the skull’s movement were cross-checked in real-time with the digital output from the VR Head Tracking application (Figure 2 and Figure 3).

Before beginning the measurement trials with the model and VR headset, the researchers ensured alignment between the starting position in degrees in the application and on the model’s goniometer. Next, the researcher prepared a data set for the study, consisting of angles in degrees and ranges of motion in three planes. These data were written on white paper sheets, with the notation facing down, and arranged on a desk in two separate columns. The second researcher randomly selected two sheets three times, in sequence: one sheet from the column with degrees, and the other with the movement to be performed. The first researcher then read the obtained data. For the model measurement, a randomly selected range of motion was 34° flexion, 45° right rotation, and 30° right lateral flexion. The second researcher performed the movements by manually replacing the skull on the model in order to obtain selected degrees. During this task, the first investigator was following the changes on the tablet live preview from the VR Head Tracking application. Subsequently, after establishing the indicated range of motion in a given movement and plane, the range obtained from the validated goniometer was noted by the researcher. The measurement on the model was performed in three independent trials.

**Scheme 1:**
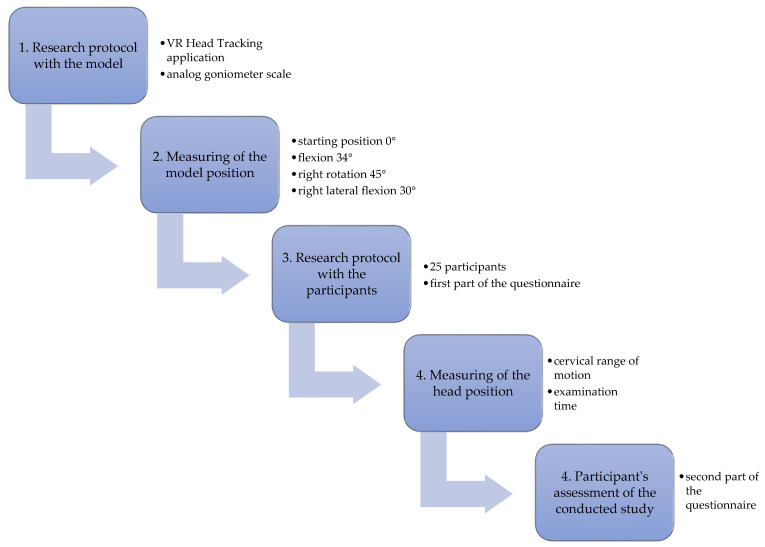
Study protocol.

In the second part of the study, we measured the range of motion in the participants’ cervical spines while also recording the duration of the entire examination procedure. Each participant was supposed to perform the given task in the same order, meaning the sequence of one’s maximal movements of: flexion, extension, left side bend, right side bend, left rotation, and right rotation. The measurement position was equal for each participant. The starting position for the examination was a seated position on a chair, with the back supported by the backrest and the lower limbs stabilized on the ground. The subject was informed that, after each movement, they should return to the starting position indicated in the application and begin the next movement from there, following the previously established sequence. The study protocol is presented in Scheme 1.

## 3. Results

**Figure 4:**
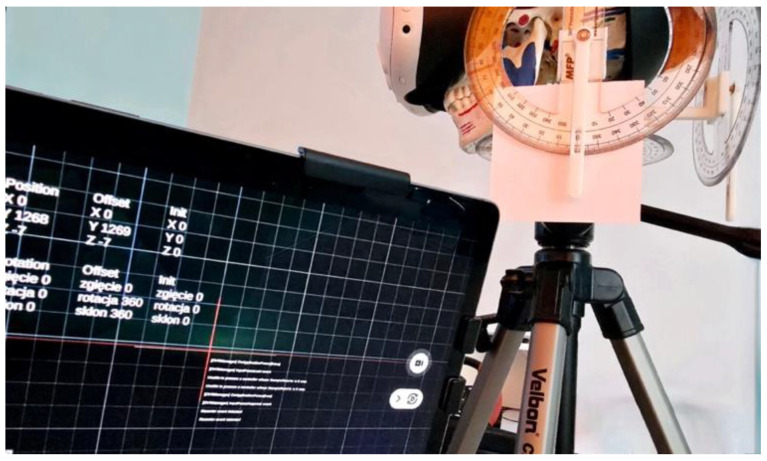
Starting position of 0° to perform the movement in the sagittal plane.

**Figure 5:**
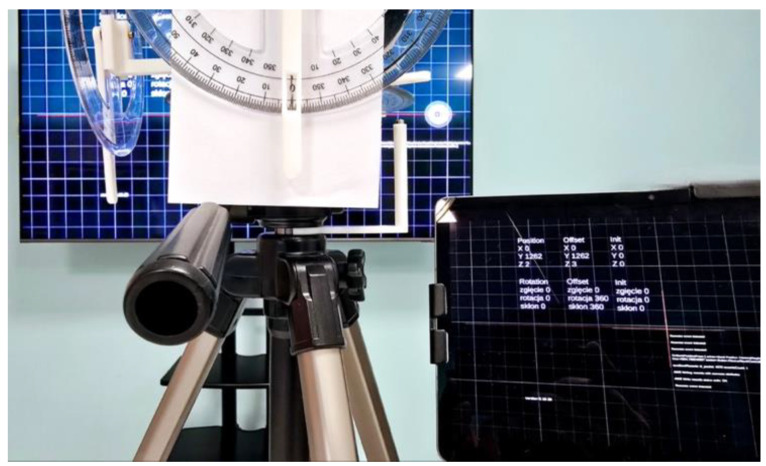
Starting position of 0° to perform the movement in the frontal and transverse planes.

**Figure 6:**
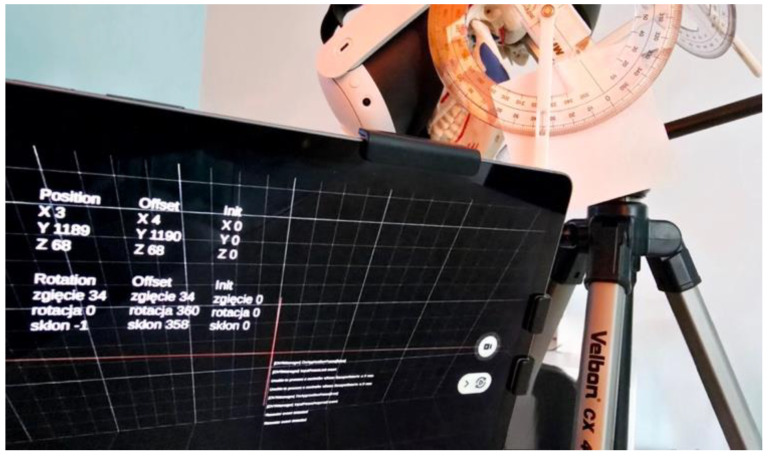
Final position for the movement in the sagittal plane: 34° of cervical spine flexion.

**Figure 7:**
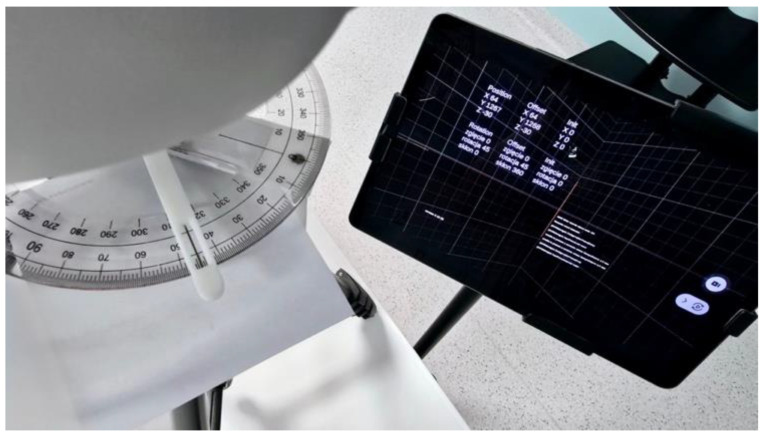
Final position for the movement in the transverse plane: 45° of cervical spine right rotation.

**Figure 8:**
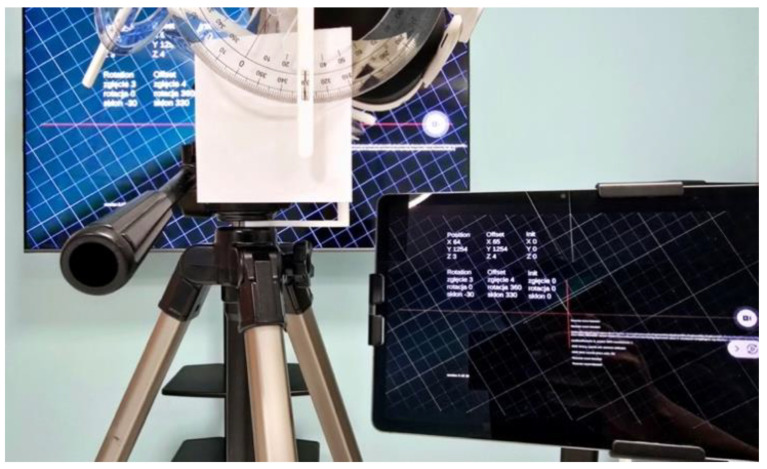
Final position of the movement in frontal plane: 30° of cervical spine right lateral flexion.

In the first part of the study, utilizing the research model, we obtained results from the VR Head Tracking application as well as readings from an analog goniometer scale. The measured angle of the model’s position, as recorded by both the application and the goniometer, was identical in all directions and planes of movement. In both cases, we recorded an angle of 0° in the starting position, followed by identical results of 34° flexion, 45° right rotation, and 30° right lateral flexion. The digital measurement result was confirmed by the range of motion measurement conducted using the classical method. Detailed results from the measurement trials are presented in Table 1 and in Figure 4, Figure 5, Figure 6, Figure 7 and Figure 8.

In the second part of the study, we read and collected data from the VR Head Tracking application immediately after completing the research protocol. For the proper interpretation of the numerical angular values from the ranges of motion and the axes of the graph, we used the following notation:

- **Flexion:** result with the “+” sign
- **Extension:** result with the “−” sign
- **Left lateral flexion:** result with the “+” sign
- **Right lateral flexion:** result with the “−” sign
- **Left rotation:** result with the “−” sign
- **Right rotation:** result with the “+” sign

The range of motion results in degrees for each movement, along with the minimum, maximum, and average values, and the duration of the testing procedure, are presented in Table 2. The last line of the table presents the cervical range of motion normative values [14]. These data are for information only and were not subject to statistical analysis, because they were read from the application to confirm its functionality.

**Chart 1:**
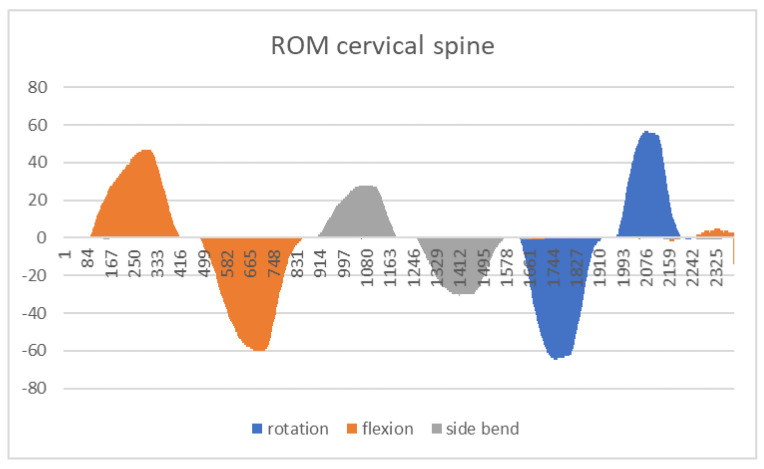
Ranges of cervical spine motion.

A sample result is also presented in graphical form in Chart 1.

From the second part of the questionnaire, which included a satisfaction survey, we recorded a 100% satisfaction level with participation in the study. All participants indicated that the study felt short in duration, the instructions were clear, and there were no difficulties in following them, nor did the procedure cause any pain. In total, 92% of the participants would choose to undergo such a study in the future, despite the fact that 88% of respondents had never before experienced a similar study using Virtual Reality. Two participants found the examination inconvenient, no one had experienced such an examination before, one person preferred traditional examination methods, and one person would not want to repeat the examination in this form. None of the participants reported any adverse effects resulting from the diagnostic procedure.

## 4. Discussion

Improving objectivity in the diagnostic process in physiotherapy is a key starting point for planning an individualized rehabilitation program. Maintaining the head in the correct position and the ability to physiologically observe the surroundings through proper visual control contribute to the smooth performance of daily activities, reduce the risk of falls, and thereby determine the individual’s independence and self-sufficiency. Treleaven and Mettelinge et al. also discussed this in their studies [26,27]. In our opinion, the necessity of searching for objective methods of assessing the cervical spine stems from the insufficient measurement accuracy of active head movements and the subjective nature of research protocols using diagnostic tools such as a measuring tape or a goniometer. Our assumptions are supported by the studies of Engelmann et al. and Valera-Calero et al. [28,29]. The results obtained from the VR Head Tracking application provide the opportunity for a more comprehensive analysis of cervical spine mobility than standard measurement methods. This is due to their presentation in both numerical and graphical forms. This approach offers specialists significantly greater interpretative potential, as previously noted in the research of Miller Spoto et al. [30].

Thanks to the 60-frame-per-second tracking of the head position in space, the application enables the additional analysis and interpretation of the obtained results, not only in terms of the range of motion and movement patterns but also the duration of movement in each direction individually. Based on the collected data, we demonstrated that the same movement performed in a given plane but in a different direction does not necessarily occur within the same timeframe. In the case of the same movement performed in opposite directions—left and right—this may result from greater joint functionality in the cervical spine, manifesting as increased mobility on one side, muscular–fascial imbalance, or hypertonia or hypotonia of specific soft tissues, particularly muscle contractures in the cervical spine and shoulder girdle region. These tissues collectively influence both the cervical spine and the shoulder. Another possible symptom is pain experienced by the subject during the movement. Using these data, we can precisely determine the severity of cervical spine dysfunctions. These relationships were described in the studies of Tsang et al. [31].

**Chart 2:**
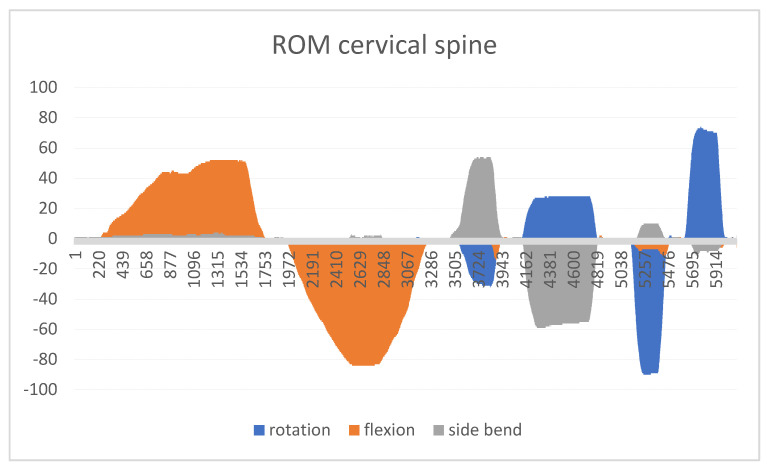
Graphical representation of range of motion from the VR Head Tracking application in the case of compensatory movements.

An additional aspect that caught our attention during the analysis of the results was the interaction of data from different planes when recording specific ranges of motion: for instance, data from movement “x” during the execution of movement “y”. Although the participants were healthy and did not report cervical spine pain, the trajectory of their head movements was not always consistent, meaning it was not parallel to the lines defined in the application. The movement trajectory in the application is outlined by lines intersecting at a 90° angle, forming a posturographic grid. The follow-up evaluation focused on the participants’ ability to maintain the principle of two parallel lines: the fixed yellow line from the application and the movable red line, which reflected the change in the participant’s head position during the test. The recording of additional movements during the tested motion may indicate that the VR Head Tracking application is not only capable of registering basic movement parameters in degrees, but also, with proper analysis of the data graph, providing information about the alignment of the performed movement with the movement pattern for the cervical spine. The alignment of the movement with the movement pattern for the upper trunk quadrant was mentioned in the research conclusions of Schuermans et al. [32]. The compensations that can be detected in this way can be observed in the following graph (Chart 2), which shows co-movements during the execution of specific movement patterns. This graph shows that, during left lateral flexion, additional left rotation and a slight extension component were registered. During right lateral flexion, compensatory right rotation occurred. Meanwhile, during rotational movements of the head, components of lateral flexion and extension appeared on the graph. The example below also suggests that the maximum range exceeds the physiological norm in the recorded measurement, compared to the generally accepted range of motion norms for the subject’s age [25]. This may also confirm that, in this case, we are dealing with an abnormal movement resulting from a compensatory mechanism.

As a result of the lack of access to such biomechanical analysis, a specialist may have an inaccurate perception of the actual range of motion of the cervical spine, based solely on their subjective feelings about the examination. The VR Head Tracking application provides the opportunity to eliminate these inaccuracies by comparing the data with the movement pattern and the expected actual range of head motion.

Based on our observations, we note that modern technology using virtual environments brings measurable benefits for diagnostics, is well tolerated by participants, and ensures full safety during the collection of data related to the cervical spine range of motion. This may suggest that, subsequently, a therapeutic approach applied in the same virtual conditions would be an equally appropriate clinical decision. Virtual Reality has been successfully applied both diagnostically and therapeutically and has been described in numerous scientific reports in recent years, as presented in the literature review conducted by Hamid et al. [33].

We are aware of the need for the further testing of such modern solutions in physiotherapy. In the near future, we plan to use the application on a larger scale and use it to diagnose cervical spine disorders in patients with various medical diagnoses.

## 5. Conclusions

1. The measurement of cervical spine range of motion using the proprietary VR Head Tracking application is reliable and objective.
2. The level of satisfaction with the diagnostic method, as well as the tolerance of the VR examination, is consistently favorable in adults.
3. The results obtained from the VR application allow for a deeper analysis of the data, beyond the angles of the range of motion.
4. The use of diagnostic methods in a Virtual Reality environment for assessing the biomechanics of the musculoskeletal system in the cervical spine is valid and can be efficiently and non-invasively performed.

## Figures and Tables

**Table 1:** The angle values obtained from the first part of the study.

Measurement	Application Result	Goniometer Result	Figure
Starting position	0°	0°	Figure 4 and Figure 5
1—flexion	34°	34°	Figure 6
2—right rotation	45°	45°	Figure 7
3—right lateral flexion	30°	30°	Figure 8

**Table 2:** Ranges of cervical spine motion and examination times.

Participant	Flexion [°]	Extension [°]	Left Side Bend [°]	Right Side Bend [°]	Left Rotation [°]	Right Rotation [°]	Examination Time [s]
1	47	60	31	28	65	57	40
2	50	67	28	34	66	71	63
3	42	73	45	45	80	80	46
4	47	79	44	45	73	75	61
5	50	49	36	35	82	63	41
6	41	53	29	23	64	60	36
7	32	43	30	33	58	54	30
8	32	43	30	33	58	54	24
9	49	65	45	45	79	80	59
10	45	59	43	43	80	80	55
11	50	79	38	45	80	80	46
12	45	59	43	43	86	80	47
13	49	79	44	43	80	80	38
14	49	76	44	38	70	67	45
15	50	80	45	45	80	80	52
16	45	76	45	45	80	79	33
17	43	80	42	40	79	79	58
18	49	76	45	38	70	67	34
19	44	61	43	39	73	74	50
20	36	51	39	35	67	66	76
21	46	28	37	39	53	60	28
22	39	71	34	40	61	67	40
23	49	77	30	44	77	79	45
24	50	80	45	45	80	74	90
25	36	52	29	31	65	63	62
**average**	**44.6**	**64.64**	**38.56**	**38.96**	**72.24**	**70.76**	**47.96**
**min**	**32**	**28**	**28**	**23**	**53**	**54**	**24**
**max**	**50**	**80**	**45**	**45**	**86**	**80**	**90**
**normative value** [25]	**50**	**80**	**45**	**45**	**80**	**80**	**-**

## Data Availability

Data are contained within the article.
